# Supplementary material for: Determination of Phenolic Compounds by Capillary Zone Electrophoresis–Mass Spectrometry
Source: Molecules. 2022 Jul 16;27(14):4540. doi: 10.3390/molecules27144540 (PMC9316225; doi:10.3390/molecules27144540)
Supplement: Supplementary file 1 [file molecules-27-04540-s001.zip › molecules-1769844-supplementary.pdf]

Determination of phenolic compounds by capillary zone electrophoresis – mass spectrometry

Ruben Szabo, Attila Gaspar*

Department of Inorganic and Analytical Chemistry, University of Debrecen, Egyetem ter 1., Debrecen 4032, Hungary

Supplementary Materials

*Corresponding author

E-mail: gaspar@science.unideb.hu

Tel: +36-30-2792889, Fax: +36-52-518660

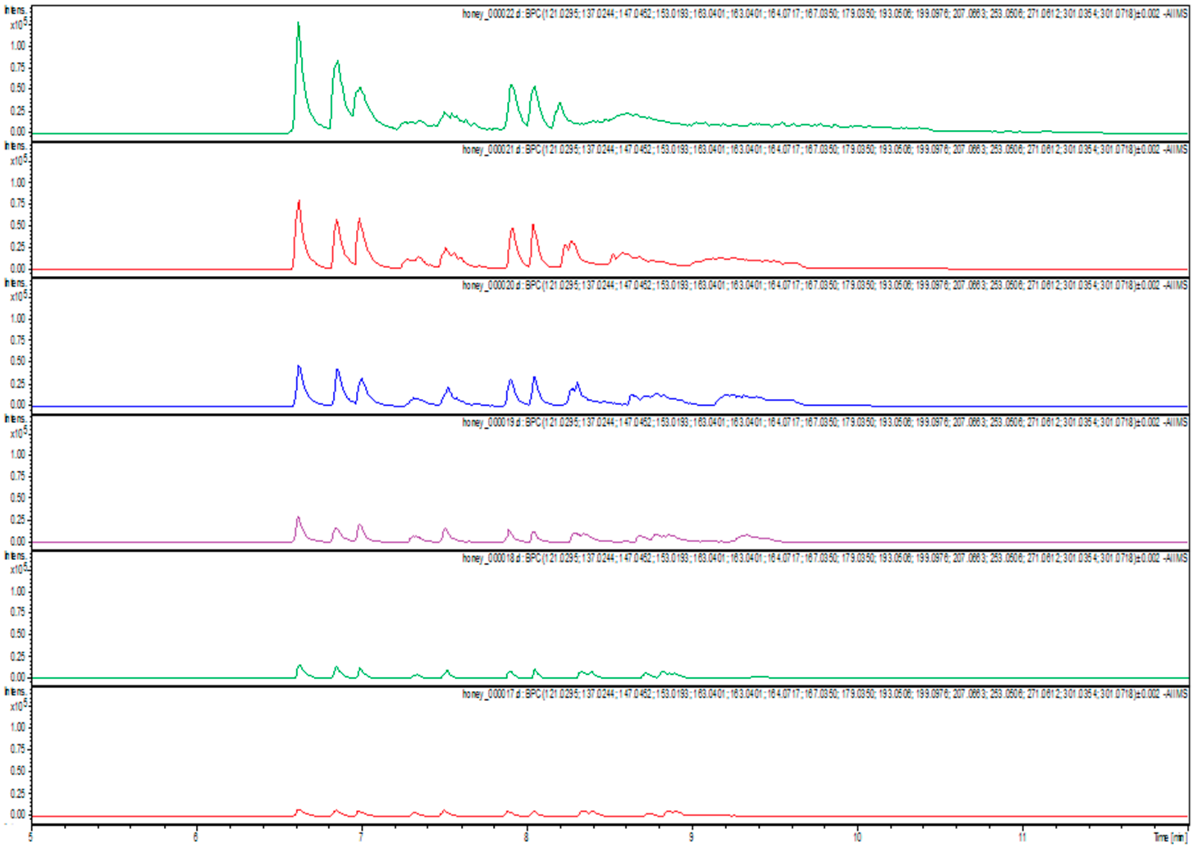

Figure S1: CZE-MS measurements of different concentration standard solution mixtures. Conditions are the same as in Figure 2a. From top to bottom, concentrations are 5, 3.33, 2.5, 1, 0.5 and 0.33 times higher for all compounds than as specified in Figure 2a.

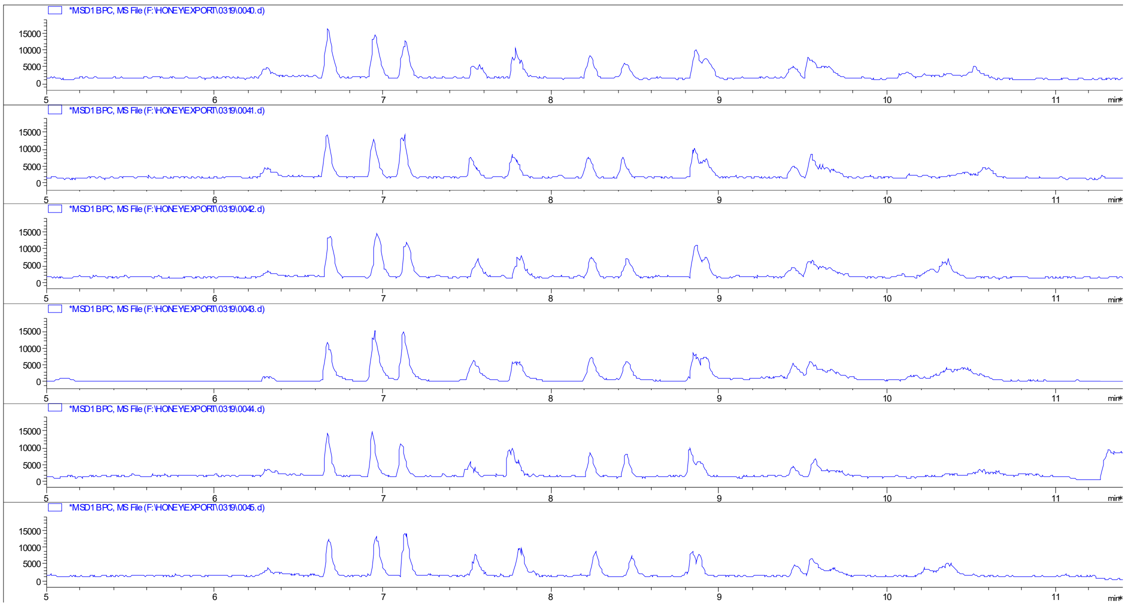

Figure S2: Six repetitions of CZE-MS measurements of standard solution mixture. Conditions are the same as in Figure 2a.
